# F199. O-GLCNAC DYSREGULATION IN SCHIZOPHRENIA CORTEX

**DOI:** 10.1093/schbul/sby017.730

**Published:** 2018-04-01

**Authors:** Toni Mueller, Anita Pinner, James Meador-Woodruff

**Affiliations:** University of Alabama at Birmingham

## Abstract

**Background:**

O-linked β-linked N-acetylglucosamine (O-GlcNAc) is a post-translational glycosylation modification with ubiquitous functions in cell biology. The attachment of GlcNAc to serine or threonine (S/T) residues transiently adorns thousands of nuclear, cytosolic, and mitochondrial proteins and modulates protein function and localization via dynamic cooperation with kinase and phosphatase enzymes. O-GlcNAc transferase (OGT) exists in complex with S/T phosphatase subunits PP1β and PP1γ and, along with the activity of O-GlcNAcase (OGA), facilitates rapid cycling between O-GlcNAcylation and phosphorylation states to serve as an “on/off switch” for substrate activation. Altered levels of OGT, OGA, and/or O-GlcNAc have been shown to influence many pathways pertinent to schizophrenia (SZ) pathophysiology. Notably, elevated O-GlcNAc and enhanced O-GlcNAcylation mediate glucose tolerance and insulin resistance which can lead to diabetes, an illness often found comorbid with SZ. Elevated O-GlcNAcylation can also produce mitochondrial abnormalities consistent with those identified in SZ. In an exploratory study of glycosylation enzyme transcript expression, our lab found OGT mRNA levels 253% higher in SZ than non-psychiatrically ill comparison (COMP) subjects (p < 0.0001). Based on this evidence, we hypothesized that OGT protein levels or the ratio of OGT:OGA enzymes are elevated in SZ brain.

**Methods:**

Expression of OGT and OGA were measured using western blots of superior temporal gyrus (STG; Brodmann Area 22) homogenates from sex- and age-matched pairs of SZ and COMP subjects (N = 17). Standard immunoblotting methods and commercially available antibodies were used to detect the targets of interest and protein levels were normalized to intralane valosin containing protein (VCP) expression; VCP expression has previously been found to be unchanged in SZ STG.

**Results:**

In the current study, we found OGA protein levels reduced 18% in SZ (p < 0.01) and SZ subjects demonstrate a trend toward increased ratios of OGT:OGA (p = 0.05). OGT was not different between groups (p = 0.77).

**Discussion:**

Our current results partially support our original hypothesis that an altered ratio of OGT:OGA may contribute to abnormalities of O-GlcNAcylation and consequent cellular metabolic abnormalities in SZ. A trend toward increased OGT:OGA along with decreased expression of OGA would produce the same functional outcome as the originally predicted OGT increase: upregulation of protein O-GlcNAcylation. Given that the mRNA study used samples of dorsolateral prefrontal cortex (DLPFC) while our protein-level measures were from STG, it is not inconceivable that potential O-GlcNAc dysregulation could arise from upregulated OGT in one brain region, but downregulated OGA in another. To elaborate on these findings, we will investigate OGT and OGA expression in the DLPFC and will assess total O-GlcNAcylation in both brain regions to determine functional consequences of altered enzyme expression.

